# The prognostic value of MELD-XI in elderly patients with ST-segment elevation myocardial infarction: an observational study

**DOI:** 10.1186/s12872-021-01862-5

**Published:** 2021-01-28

**Authors:** Song-jian He, Jian-xin Weng, Hai-jun Chen, Hua-qiu Li, Wen-qin Guo, Qian Cao, Shuai Xu, Hong-bing Yan, Chang-nong Peng

**Affiliations:** 1 Department of Coronary Heart Disease, Fuwai Hospital Chinese Academy of Medical Sciences, Shenzhen, Langshan Road 12, Shenzhen, 518000 China; 2 Department of Coronary Heart Disease, Peking University Shenzhen Hospital, 1120 Lianhua St, Futian District, Shenzhen, 518000 China

**Keywords:** MELD-XI, TIMI risk score, ST-segment elevation myocardial infarction, Percutaneous coronary intervention

## Abstract

**Background:**

The model for end-stage liver disease excluding international normalized ratio (MELD-XI) is a simple score for risk assessment. However, the prognostic role of MELD-XI and its additional value to current risk assessment in elderly patients with ST-segment elevation myocardial infarction (STEMI) undergoing percutaneous coronary intervention (PCI) is uncertain.

**Methods:**

In all, 1029 elderly patients with STEMI undergoing PCI were consecutively included and classified into three groups according to the TIMI risk score: low-risk (≤ 3, n = 251); moderate-risk (4–6, n = 509); and high-risk (≥ 7, n = 269) groups. Multivariate analysis was performed to identify risk factors for adverse events.

**Results:**

The overall in-hospital mortality was 5.3% and was significantly higher in the high-risk group (1.2% vs. 3.3% vs. 13.0%, p < 0.001). The optimal cut-off of the TIMI risk score and MELD-XI for in-hospital death was 7 and 13, respectively. MELD-XI was associated with in-hospital (adjusted odds ratio = 1.09, 95% CI = 1.04–1.14, p = 0.001) and one-year (adjusted hazard ratio = 1.05, 95% CI = 1.01–1.08, p = 0.005) mortality independently of the TIMI risk score. Combining TIMI risk score and MELD-XI exhibited better predictive power for in-hospital death than TIMI risk score (area under the curve [AUC] = 0.810 vs. 0.753, p = 0.008) or MELD-XI alone (AUC = 0.810 vs. 0.750, p = 0.018). Patients with TIMI risk score ≥ 7 and MELD-XI ≥ 13 had the worst prognosis.

**Conclusion:**

MELD-XI could be considered as a risk-stratified tool for elderly patients with STEMI undergoing PCI. It had an additive prognostic value to TIMI risk score.

## Introduction

Early diagnosis and intervention have dramatically improved the prognosis of ST-segment elevation myocardial infarction (STEMI) [1, 2]. However, the mortality from STEMI remains high in elderly patients. Epidemiological data have indicated that the estimated one-year mortality in elderly patients with STEMI undergoing percutaneous coronary intervention (PCI) is 10% and continues to increase with age [3–5]. The Thrombolysis In Myocardial Infarction (TIMI) risk score is a guideline-recommended, risk-stratified tool for STEMI patients [6]. Nevertheless, this score is not established specially for elderly patients. Previous studies have shown that the area under the curve (AUC) of TIMI risk score was only 0.71 for predicting in-hospital mortality and 0.67 for 30-day mortality [7, 8] in elderly patients with STEMI. Therefore, improving the predictive accuracy is necessary for these patients.

Aging represents the progressive functional decline in a variety of organ systems that occurs with advancing time [9]. Renal and liver dysfunction were common in elderly patients with myocardial infarction, which were associated with poor prognosis [10, 11]. The model for end-stage liver disease excluding international normalized ratio (MELD-XI) score is a novel and simple score to simultaneously assess liver and kidney function [12]. This scoring model exhibited excellent predictive power in critically ill patients [13, 14]. However, the association of MELD-XI with adverse events has not been reported in STEMI patients. Therefore, this study was performed to investigate the prognostic value of MELD-XI in elderly patients with STEMI undergoing PCI and determine whether adding MELD-XI to the TIMI risk score enhances the prediction of in-hospital and one-year death.

## Methods

### Study population

This was a single-center cohort study conducted at Fuwai Hospital Chinese Academy of Medical Sciences, Shenzhen. A total of 1137 elderly patients (≥ 60 years) with STEMI undergoing PCI were retrospectively screened from January 2010 to December 2018. STEMI was defined according to the European Society of Cardiology guidelines [15]. 108 were excluded for the following reasons: hospital stay < 24 h (n = 23), malignancy (n = 19), aortic dissection (n = 6), requiring coronary artery bypass grafting (n = 31) and without admission data on bilirubin (n = 29). The study protocol was approved by the Ethics Committee of our hospital (SP2017016), and the need for informed consent was waived given the retrospective nature of the analysis.

### Measurement and data collection

Serum creatinine and bilirubin were measured at admission. Left ventricular ejection fraction (LVEF) was evaluated by echocardiography within 24 h after admission. The demographic and clinical characteristics were collected from electronic medical records by one researcher and randomly checked by another researcher. The TIMI risk score for patients with STEMI was calculated based on the following characteristics: age; systolic blood pressure (SBP); heart rate; Killip classification; weight; history of angina, diabetes, and/or hypertension; presence of anterior myocardial infarction and/or left bundle branch block presentation; and time to treatment [16]. The MELD-XI score was calculated by using the following formula: 5.11 × (ln total bilirubin, mg/dL) + 11.76 × (ln creatinine, mg/dL) + 9.44 [17].

### Follow-up and endpoints

Each patient was followed-up via telephonic interview for one year after a diagnosis of STEMI. We also reviewed the outpatient and re-admission records for possible adverse events. The primary endpoints were in-hospital and one-year all-cause mortality. In addition, the incidence of stroke, dialysis, acute heart failure, and target vessel revascularization (TVR) during the hospitalization period were defined as major adverse clinical events (MACEs).

### Statistical analysis

All statistical analyses were performed using SPSS 22.0 (Inc., Chicago, Illinois). Continuous data were expressed as mean ± SD or median (IQR) and compared using the two independent samples *t*-test or Wilcoxon rank-sum test. Categorical data were shown as frequency and percentage, and compared using the chi-squared test. The predictive value was assessed by AUC obtained through receiver operator characteristic (ROC) curves. The AUC was compared using nonparametric tests [18]. In addition, the optimal cut-off for predicting in-hospital death was recorded. The risk factors of in-hospital and one-year mortality were determined by logistic regression analysis and Cox regression analysis, respectively. All significant variables in the univariate analysis, except the elements of the TIMI risk score, were included in the multivariate analysis. The Kaplan–Meier curve for one-year mortality was plotted and compared using the log-rank test. For all comparisons, p < 0.05 was considered to indicate statistical significance.

## Results

In all, 1029 STEMI patients were included, and were divided into three groups according to the TIMI risk score: low risk (≤ 3, n = 251); moderate risk (4–6, n = 509); and high risk (≥ 7, n = 269). The clinical characteristics according to different risk status were showed in Table 1. Patients in the high-risk group were more likely to be female. The high-risk group showed higher serum creatinine, alanine transaminase (ALT), and total bilirubin levels and lower hemoglobin and LVEF than the other two groups. The rate of intra-aortic balloon pump (IABP) placement was higher in patients with a high TIMI risk score. In addition, prolonged hospitalization was observed in these patients.

**Table 1:** Clinical characteristics according to different risk status

Variable	Low-risk group (≤ 3, n = 251)	Moderate-risk group (4–6, n = 509)	High-risk group (≥ 7, n = 269)	P value
Age, years	64.0 ± 4.2	71.6 ± 6.5	73.3 ± 6.5	< 0.001
Female sex, n (%)	36 (14.3)	136 (26.7)	89 (33.1)	< 0.001
Diabetes mellitus, n (%)	58 (23.1)	142 (27.9)	83 (30.9)	0.136
Hypertension, n (%)	147 (58.6)	322 (63.3)	156 (58.0)	0.258
Weight, kg	67.8 ± 9.7	61.4 ± 9.8	59.5 ± 9.3	< 0.001
Time to admission > 4 h, n (%)	181 (72.1)	444 (87.2)	244 (90.7)	< 0.001
Anterior myocardial infarction, n (%)	78 (31.1)	224 (44.0)	169 (62.8)	< 0.001
SBP, mmHg	127.2 ± 17.7	126.1 ± 20.8	110.7 ± 26.4	< 0.001
DBP, mmHg	75.6 ± 11.2	73.4 ± 12.4	67.6 ± 14.4	< 0.001
Heart rate, bpm	76.4 ± 12.5	77.5 ± 13.1	86.3 ± 21.0	< 0.001
Killip II–IV, n (%)	16 (6.4)	145 (28.5)	208 (77.3)	< 0.001
Serum creatinine, mg/dL	1.0 (0.8,1.1)	1.0 (0.8,1.3)	1.2 (0.9,1.5)	< 0.001
Hemoglobin, g/L	133.1 ± 14.0	126.6 ± 15.7	124.4 ± 18.8	< 0.001
Creatine kinase MB, U/L	68.7 (30.4,135.1)	57.8 (23.8,135.2)	62.8 (21.3,159.6)	0.290
ALT, U/L	39.0 (28.4,54.2)	42.0 (28.1,65.0)	46.1 (31.0,77.8)	0.001
Total bilirubin, mg/dL	1.0 ± 0.4	1.0 ± 0.4	1.1 ± 0.6	0.029
LVEF, %	55.0 ± 9.8	52.5 ± 11.0	46.8 ± 12.2	< 0.001
IABP, n (%)	10 (4.0)	55(10.8)	76 (28.3)	< 0.001
Thrombus aspiration, n (%)	94 (37.5)	156(30.6)	82 (30.5)	0.130
Treated vessel, n (%)				
Any left main	9 (3.6)	25 (4.9)	19 (7.1)	0.185
Multi-vessel	21 (8.4)	62 (12.2)	27 (10.0)	
Others	221 (88.0)	422 (82.9)	223 (82.9)	
Number of stents	1.3 ± 0.7	1.5 ± 0.8	1.4 ± 0.8	0.073
Hospital stay (days)	6 (5,7)	7 (6,10)	9 (6,14)	< 0.001
In-hospital events				
Death	3 (1.2)	17 (3.3)	35 (13.0)	< 0.001
Cardiac death	3 (1.2)	13 (2.6)	25 (9.7)	< 0.001
MACEs	14 (5.6)	55 (10.8)	65 (24.2)	< 0.001

SBP, systolic blood pressure; DBP, diastolic blood pressure; ALT, alanine transaminase; LVEF, left ventricular ejection fraction; IABP, intra-aortic balloon pump; MACEs, major adverse clinical events

**Fig. 1:**
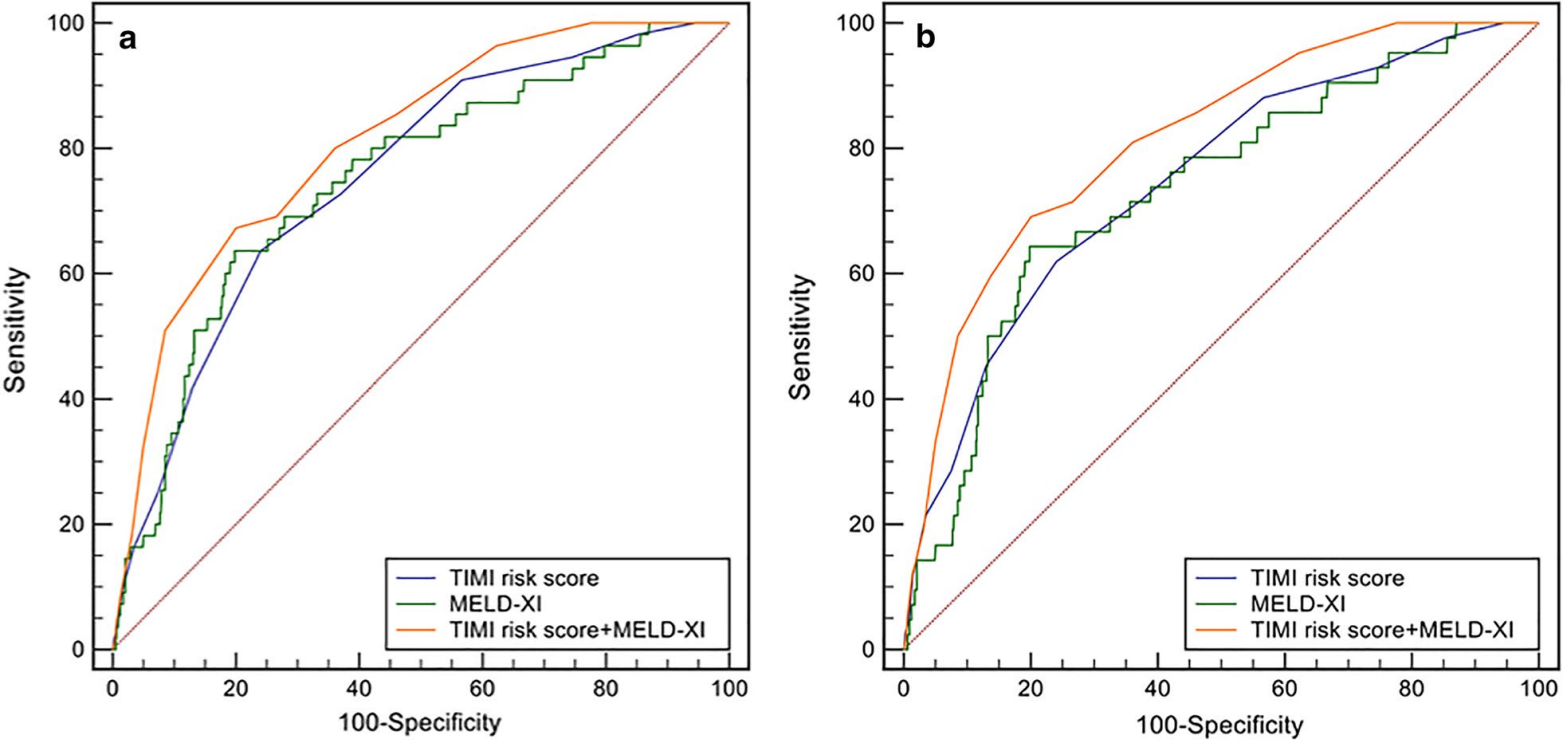
ROC curve for in-hospital mortality (**a** all-cause death, **b** cardiac death)

55 (5.3%) patients died while in hospital, 42 of which died from cardiac disease. Other causes of death included infection, cerebral hernia and multiple organ failure. A higher in-hospital all-cause mortality (1.2% vs. 3.3% vs. 13.0%, p < 0.001), cardiac mortality (1.2% vs. 2.6% vs. 9.7%, p < 0.001) and MACEs (5.6% vs. 10.8% vs. 24.2%, p < 0.001) were found in patients with high TIMI risk score than the low- and medium-risk groups. The ROC curve showed that MELD-XI ≥ 13 had a sensitivity of 63.6% and specificity of 80.2% for predicting in-hospital all-cause death (AUC = 0.750, 95% CI = 0.684–0.816, p < 0.001, Fig. 1). The AUC of TIMI risk score was 0.753 and the optimal cut-off was 7. No statistically significant difference was found on comparing their discriminatory abilities.

Multivariate logistic regression analysis indicated that MELD-XI was independently associated with in-hospital all-cause death even after adjusting for TIMI risk score, log(creatine kinase MB), ALT > 40 U/L, LVEF, and IABP treatment (OR = 1.09, 95% CI = 1.04–1.14, p = 0.001, Table 2). A similar result was seen when we included MELD-XI ≥ 13 instead of MELD-XI as a continuous variable in model 2 (OR = 3.83, 95% CI = 1.99–7.40, p < 0.001, Table 2). Therefore, we added four points into the TIMI risk score when MELD-XI ≥ 13 according to the adjusted OR. Combining TIMI risk score and MELD-XI exhibited better predictive power for in-hospital all-cause death than the TIMI risk score (AUC: 0.810 vs. 0.753, p = 0.008, Fig. 1a) or MELD-XI alone (AUC: 0.810 vs. 0.750, p = 0.018, Fig. 1a). Similar result was found in cardiac mortality (Fig. 1b).

**Table 2:** Multivariable logistic regression analysis for in-hospital death

Clinical variables	OR	95% CI	P-value
Model 1			
TIMI risk score	1.22	1.07,1.40	0.004
MELD-XI	1.09	1.04,1.14	0.001
log(creatine kinase MB)	1.44	0.79,2.61	0.236
ALT > 40 U/L	1.48	0.68,3.20	0.326
LVEF	0.98	0.96,1.01	0.178
IABP	3.69	1.85,7.36	< 0.001
Model 2			
TIMI risk score	1.23	1.07,1.41	0.003
MELD-XI ≥ 13	3.83	1.99,7.40	< 0.001
log(creatine kinase MB)	1.63	0.88,3.01	0.122
ALT > 40 U/L	1.28	0.59,2.76	0.527
LVEF	0.98	0.96,1.01	0.226
IABP	3.59	1.79,7.19	< 0.001

OR, odds ratio; CI; confidence interval; TIMI, Thrombolysis In Myocardial Infarction; MELD-XI, Model for End-stage Liver Disease excluding INR; ALT, alanine transaminase; LVEF, left ventricular ejection fraction; IABP, intra-aortic balloon pump

In total, 109 (10.6%) patients died during the one-year follow-up period. Multivariate Cox regression analysis showed that MELD-XI was a predictor for one-year mortality independent of the TIMI risk score (HR = 1.17, 95% CI = 1.07–1.27, p < 0.001, Table 3). Other significant risk factors included LVEF and IABP treatment.

**Table 3:** Univariate and multivariate Cox survival analysis for one-year mortality

Clinical variables	Univariate analysis		Multivariable analysis		
	HR	p-value	HR	95% CI	p-value
TIMI risk score	1.35	< 0.001	1.17	1.07,1.27	< 0.001
MELD-XI	1.09	< 0.001	1.05	1.01,1.08	0.005
Female sex	1.15	0.500			
Diabetes mellitus	1.84	0.002	1.40	0.94,2.10	0.101
Hypertension	1.12	0.558			
Anemia	1.38	0.141			
log(creatine kinase MB)	1.33	0.125			
ALT > 40 U/L	1.84	0.004	1.38	0.89,2.13	0.154
LVEF	0.94	< 0.001	0.97	0.95,0.99	< 0.001
IABP	6.00	< 0.001	2.32	1.45,3.73	< 0.001
Thrombus aspiration	1.04	0.864			
LMCA intervention	1.75	0.109			

TIMI, Thrombolysis In Myocardial Infarction; MELD-XI, Model for End-stage Liver Disease excluding INR; ALT, alanine transaminase; LVEF, left ventricular ejection fraction; IABP, intra-aortic balloon pump; LMCA, left main coronary artery

**Fig. 2:**
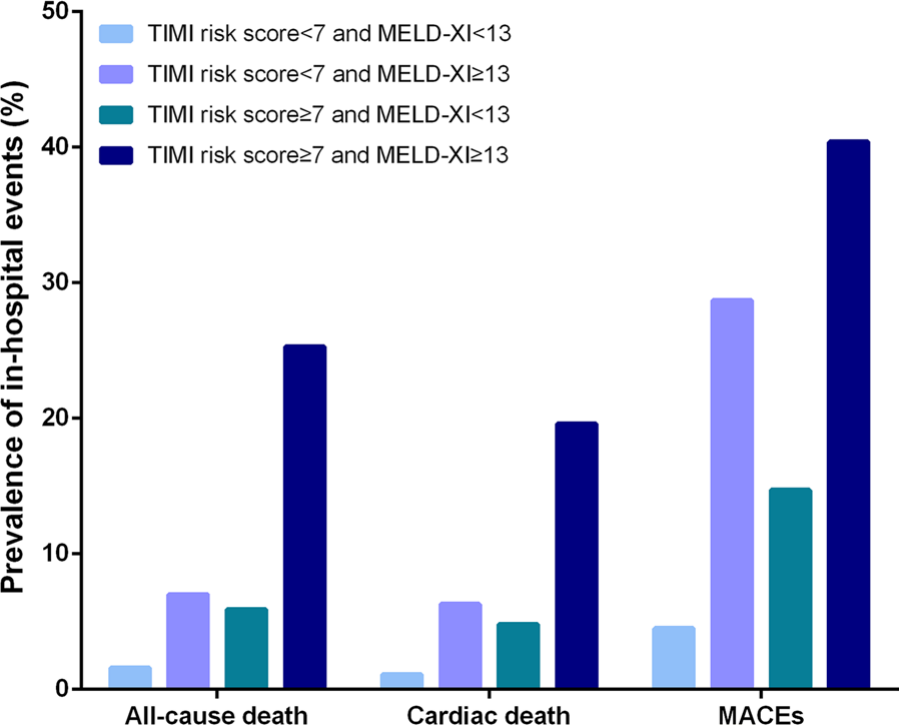
Prevalence of in-hospital events according to different levels of MELD-XI and TIMI risk scores

**Fig. 3:**
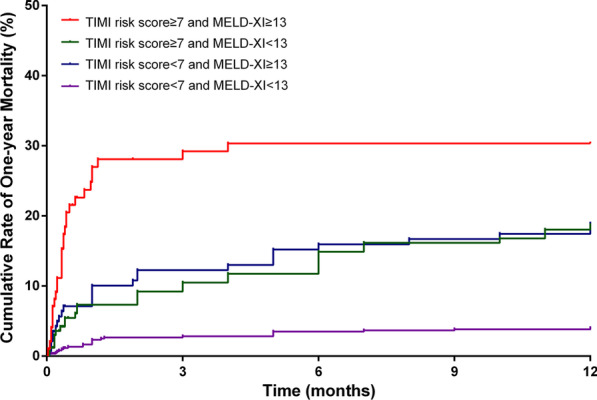
Kaplan–Meier curve for 1-year mortality

Patients were reclassified into four groups according to the cut-off of TIMI risk score and MELD-XI: TIMI risk score ≥ 7 and MELD-XI ≥ 13 (n = 99), TIMI risk score ≥ 7 and MELD-XI < 13 (n = 170), TIMI risk score < 7 and MELD-XI ≥ 13 (n = 143), and TIMI risk score < 7 and MELD-XI < 13 (n = 617). The in-hospital all-cause mortality (25.3%), cardiac mortality (19.6%) and incidence of MACEs (40.4%) were highest in the group of patients with TIMI risk score ≥ 7 and MELD-XI ≥ 13 (both p < 0.001, Fig. 2). The Kaplan–Meier curve showed that a higher accumulative one-year mortality was found in patients with TIMI risk score ≥ 7 and MELD-XI ≥ 13 (log-rank = 98.35, p < 0.001, Fig. 3). Multivariate analysis revealed that compared with TIMI risk score < 7 and MELD-XI < 13, TIMI risk score ≥ 7 and MELD-XI ≥ 13 were independently associated with in-hospital (OR = 11.06, 95% CI = 4.38–27.96, p < 0.001, Table 4), cardiac (OR = 14.09, 95% CI = 4.59–43.27, p < 0.001, Table 4) and one-year mortality (HR = 4.20, 95% CI = 2.24–7.87, p < 0.001, Table 4).

**Table 4:** Unadjusted and adjusted OR/HR of different risk

	TIMI risk score ≥ 7 and MELD-XI ≥ 13		TIMI risk score ≥ 7 and MELD-XI < 13		TIMI risk score < 7 and MELD-XI ≥ 13	
	OR/HR (95% CI)	P	OR/HR (95% CI)	P	OR/HR (95% CI)	P
In-hospital all-cause death						
Model 1: unadjusted	20.51 (9.48,44.38)	< 0.001	3.79 (1.55,9.27)	0.003	4.56 (1.86,11.19)	0.001
Model 2: multivariate adjusted*	11.06 (4.38,27.96)	< 0.001	2.83 (1.06,7.57)	0.038	3.63 (1.33,9.87)	0.012
In-hospital cardiac death						
Model 1: unadjusted	21.09 (8.53,52.18)	< 0.001	4.34 (1.55,12.14)	0.005	5.87 (2.15,16.04)	0.001
Model 2: multivariate adjusted*	14.09 (4.59,43.27)	< 0.001	3.71 (1.16,11.91)	0.027	5.56 (1.74,17.78)	0.004
One-year death						
Model 1: unadjusted	9.58 (5.57,16.46)	< 0.001	4.94 (2.89,8.46)	< 0.001	5.10 (2.93,8.89)	< 0.001
Model 2: multivariate adjusted#	4.20 (2.24,7.87)	< 0.001	3.48 (1.95,6.19)	< 0.001	3.44 (1.88,6.32)	< 0.001

TIMI risk score < 7 and MELD-XI < 13 as reference

TIMI, Thrombolysis In Myocardial Infarction; MELD-XI, Model for End-stage Liver Disease excluding INR; ALT, alanine transaminase; LVEF, left ventricular ejection fraction; IABP, intra-aortic balloon pump; LMCA, left main coronary artery

^*^Adjusted for log(creatine kinase MB), ALT > 40 U/L, LVEF, and IABP

^#^Adjusted for diabetes mellitus, ALT > 40 U/L, LVEF, and IABP

## Discussion

The present study showed that MELD-XI was an independent risk factor for in-hospital and one-year mortality after adjusting for TIMI risk score and other potential confounding factors in elderly patients with STEMI undergoing PCI. To our best knowledge, this study is the first to explore the additional prognostic value of MELD-XI to TIMI risk score. Combining TIMI risk score and MELD-XI exhibited better predictive power for in-hospital death than the TIMI risk score or MELD-XI alone. Patients with TIMI risk score ≥ 7 and MELD-XI ≥ 13 had the worst prognosis.

The TIMI risk score was first developed in the era of thrombolytic therapy and has high discriminative ability for adverse events in STEMI patients [16]. Wei et al. reported that the AUC of the TIMI risk score for predicting in-hospital mortality was 0.803 [19]. In addition, its discriminative ability was adequate for clinical application in STEMI patients undergoing primary PCI (c-statistic, > 0.8) [20]. Accordingly, this score was also recommended for early risk assessment in STEMI patients in the era of primary PCI [6]. However, its predictive power is insufficient in elderly patients. A previous study showed that the prognostic discrimination and calibration of TIMI risk score for the 30-day mortality was not high in elderly patients with STEMI (AUC = 0.67) [7]. Similar results were found in female patients with STEMI [8]. In our study, the AUC of TIMI risk score for predicting in-hospital death was 0.753. Therefore, it is necessary to identify other risk factors to improve the discriminative ability of TIMI risk score.

The MELD score consists of serum creatinine and bilirubin levels and the international normalized ratio (INR), which is established for patients with end-stage liver disease [21]. MELD-XI excluding INR is a simpler risk model that can simultaneously assess renal and liver function [12]. It has already been validated in other conditions. In patients with heart failure, MELD-XI was related to increased risk of death at the one-year follow-up [22]. The AUC of MELD-XI for predicting adverse events at the one-year follow-up was 0.69 in patients undergoing tricuspid annuloplasty [23]. In critically ill patients, MELD-XI represented organ failure and was associated with in-hospital mortality [24].

Elevated serum creatinine and bilirubin are established risk factors for poor prognosis in STEMI patients [25, 26]. Although MELD is reportedly a predictor of long-term survival in patients with acute coronary syndrome undergoing PCI, the prognostic value of MELD-XI is not yet clear [27]. Therefore, we carried out this study, the results of which showed that MELD-XI has similar predictive power as TIMI risk score in elderly patients with STEMI undergoing PCI. In addition, MELD-XI was associated with in-hospital and 1-year mortality even after adjusting for TIMI risk score and other confounding factors. Renal and hepatic function are not included elements in the TIMI risk score. Accordingly, the combination of MELD-XI and TIMI risk scores showed high predictive power for in-hospital and one-year mortality, which could be a potentially useful tool for risk assessment in elderly patients with STEMI undergoing PCI.

## Limitations

The present study has some limitations. First, clinical variables were limited owing to the retrospective study design. Second, although multivariate analysis was conducted, residual confounders might have still affected our results. Third, this study was conducted in elderly patients with STEMI undergoing PCI; therefore, it remains to be seen whether these conclusions can be extrapolated to other age groups. Finally, this study was a single-center study and thus external validity of results has not been measured.

## Conclusion

Our study demonstrated that MELD-XI could predict in-hospital and one-year mortality in elderly patients with STEMI undergoing PCI. The discriminative ability of MELD-XI for in-hospital death was equal to that of the TIMI risk score. MELD-XI could provide additional prognostic value to the TIMI risk score. Therefore, it may be clinically useful to combine the MELD-XI and TIMI risk scores for risk assessment in elderly patients with STEMI undergoing PCI.

## Data Availability

The database used and/or analyzed for this study available from the corresponding author on reasonable request (Chang-nong Peng; Email: 819650730@qq. com).

## Abbreviations

MELD-XI: The model for end-stage liver disease excluding international normalized ratio
TIMI: Thrombolysis In Myocardial Infarction
STEMI: ST-segment elevation myocardial infarction
PCI: Percutaneous coronary intervention
AUC: Area under the curve
LVEF: Left ventricular ejection fraction
SBP: Systolic blood pressure
TVR: Target vessel revascularization
MACEs: Major adverse clinical events
ROC: Receiver operator characteristic
ALT: Alanine transaminase
IABP: Intra-aortic balloon pump
INR: International normalized ratio
DBP: Diastolic blood pressure
OR: Odds ratio
CI: Confidence interval
LMCA: Left main coronary artery
